# Changing Trends in Use of Laparoscopy: A Clinical Audit

**DOI:** 10.1155/2014/562785

**Published:** 2014-12-04

**Authors:** Ritu Khatuja, Geetika Jain, Sumita Mehta, Nidhi Arora, Atul Juneja, Neerja Goel

**Affiliations:** ^1^Department of Obstetrics and Gynaecology, University College of Medical Sciences and Guru Teg Bahadur Hospital, Dilshad Garden, Delhi 110095, India; ^2^National Institute of Medical Statistics (ICMR), Ansari Nagar, Delhi, India

## Abstract

*Aim.* To find out the changing trends in indications for use of laparoscopy for diagnostic or operative procedures in gynaecology. *Methods.* This was a clinical audit of 417 women who underwent laparoscopic procedures over a period of 8 years from January 2005 to December 2012 in the Department of Obstetrics and Gynaecology at a tertiary care centre in Delhi. *Results.* A total of 417 diagnostic and operative laparoscopic procedures were performed during the period from January 2005 to December 2012. Out of 417 women, 13 women were excluded from the study due to inadequate data. 208 (51.4%) women had only diagnostic laparoscopy whereas 196 (48.6%) patients had operative laparoscopy after the initial diagnostic procedure. Change in trend of diagnostic versus operative procedures was observed from 2005 to 2012. There was increase in operative procedures from 10 (37.03%) women in 2005 as compared to 51 (73.91%) in 2012. The main indication for laparoscopy was infertility throughout the study period (61.38%), followed by chronic pelvic pain (CPP) (11.38%) and abnormal uterine bleeding (AUB) (9.4%). *Conclusion.* Over the years, there has been a rise in the rate of operative laparoscopy. Though the indications for laparoscopy have remained almost similar during the years, laparoscopy for diagnosis and treatment of CPP and AUB has now increased.

## 1. Introduction

Laparoscopy is a revolution in gynaecological surgery because of being safe and less invasive. But initially its use in gynaecology was restricted to the diagnosis of infertility and sterilization procedures. Gradually with time and increasing expertise it is being used as the diagnostic as well as the therapeutic modality in different gynaecological problems [1–3]. Today, laparoscopy is one of the most common surgical procedures performed by gynaecologists. For procedures like endometriosis or ovarian cysts it has become the treatment of choice. So, we did a clinical audit on all cases that underwent laparoscopy over 8-year period (2005–2012) in a single gynaecological unit.

The objective of the study was to find out the changing trends in indications for use of laparoscopy for diagnostic or operative procedures in gynaecology.

## 2. Methods

All the cases of laparoscopy performed from 2005 to 2012 in single unit of Department of Obstetrics and Gynaecology, at a tertiary care centre in Delhi, were retrospectively analyzed. All the cases that underwent laparoscopy for any pelvic pathology were included. Patients with incomplete data were excluded. Laparoscopy was done with the usual technique under general anaesthesia with proper informed consent. The indications and intraoperative findings were noted and evaluated in the study. Frequency distribution and percentages were calculated.

## 3. Results

**Figure 1:**
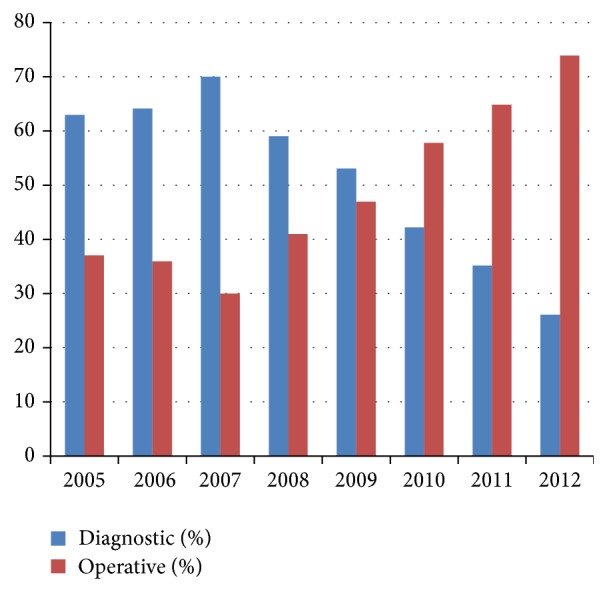
Changes in the trend of laparoscopic procedures, diagnostic versus operative from 2005 to 2012.

A total of 417 cases of laparoscopy were analyzed. Age group of the women varied from 25 years to 45 years in the study group. Thirteen cases were excluded from the study due to incomplete data. Out of 404 cases of laparoscopy, 208 (51.4%) patients had only diagnostic laparoscopy whereas in 196 (48.6%) patients operative procedures were carried out. In 2005, only 37.03% laparoscopies were operative whereas it increased to 73.91% in 2012 (Table 1, Figure 1).

**Figure 2:**
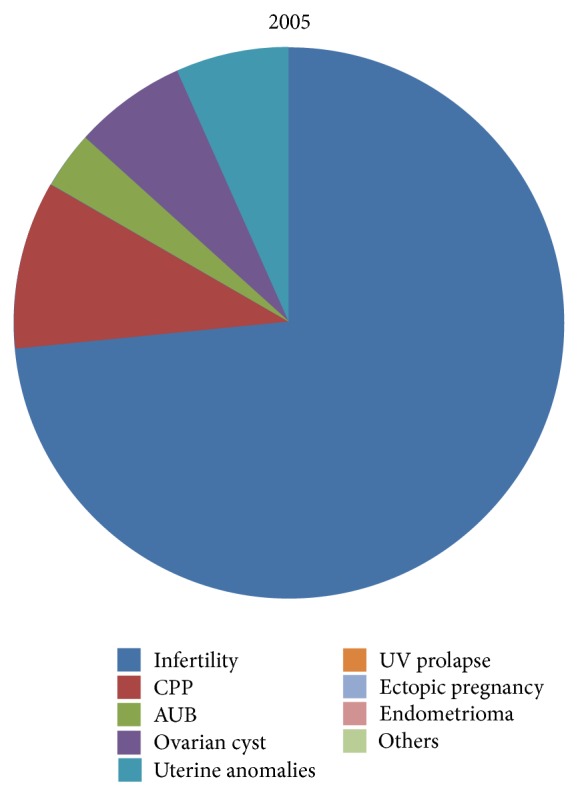
Various pelvic pathologies as an indication for laparoscopy in 2005.

**Figure 3:**
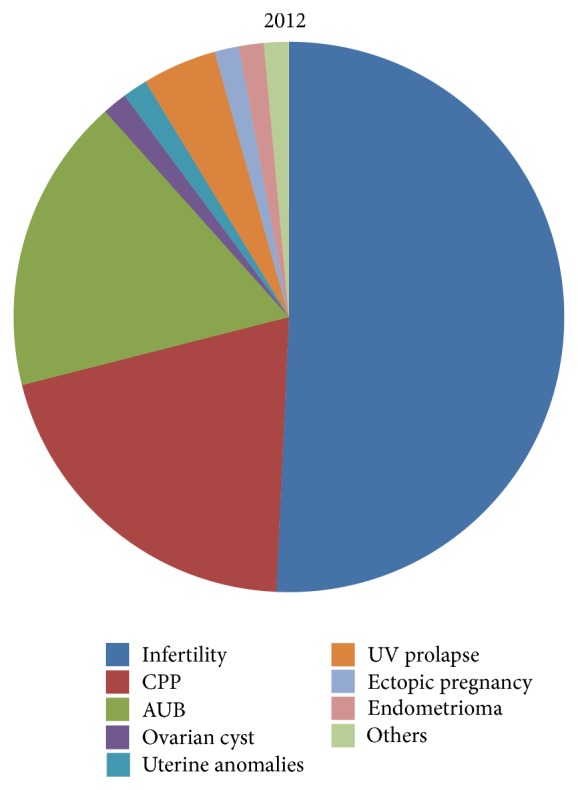
Various pelvic pathologies as an indication for laparoscopy in 2012.

The main indication for laparoscopy was infertility throughout the study period (61.38%), followed by CPP (11.38%), AUB (9.4%). In 2005 infertility was an indication in 81.4% cases, CPP in 3.7%, and AUB in 3.7% cases only (Table 2, Figure 2). In 2012, laparoscopy was done in 50.7% of women with infertility, in 20.28% with CPP, and in 17.39% with AUB. Laparoscopy for other indications like UV prolapse (4.3%) and cystectomy has also increased (Table 2, Figure 3).

## 4. Discussion

Laparoscopy has become the most common procedure in recent setup. It is now a major diagnostic as well as therapeutic modality for infertility, endometriosis, CPP, and benign ovarian tumours. For surgeries like hysterectomy, lymphadenectomy, and oncologic procedures, its use is also rapidly increasing.

Our study demonstrates an increase in operative laparoscopic surgery over 8 years in our institute which is similar to the study by Twijnstra et al. and Dhaliwal et al.; but a study by Jarrell showed that though there was a decrease in the rate of diagnostic laparoscopy there was no complementary increase in operative procedure [4–6]. In 2005 there were more cases of diagnostic laparoscopy (62.96%) as compared to operative procedures (37.04%) while in 2012 the ratio has almost reversed (diagnostic: 26.08%, operative: 73.91%). This shift in increased operative laparoscopies can be explained by the fact that as we have better noninvasive modalities for diagnosis of conditions such as AUB or ovarian cysts laparoscopy is reserved for therapeutic purposes [8]. The shift from diagnostic to therapeutic use of laparoscopy also has been facilitated by better endoscopic devices and the increase in skills of the surgeons. Laparoscopy has given in this new era of surgery less postoperative pain, early recovery to work, and in totality better patient compliance. This further decreases the cost of the treatment and so operative laparoscopy is an economically feasible option.

Our study also highlights the fact that though infertility has remained the most common indication for laparoscopy, there has been increased use of laparoscopy for chronic pelvic pain, abnormal uterine bleeding, and uterovaginal prolapse. A study by Drozgyik et al. suggests that laparoscopy is an essential method for the diagnosis and management of CPP whereas according to a study by Cox et al. laparoscopy does not appear to affect either pain symptom or quality of life in women with CPP [7, 8]. As our study is only a clinical audit we do not have long term follow-up of these women. But definitely over the years, our sensitivity has increased towards the problems of women and factors affecting their quality of life and laparoscopy as a diagnostic as well as therapeutic tool under these circumstances is remarkable. The basic advantage of laparoscopy is mainly in visualizing the pathology and then treating it in the same sitting, especially in cases of infertility, ovarian cyst, and adhesions.

The procedures like laparoscopic hysterectomy have not become very common in our study group but many studies show its increased use in gynaecology [9]. According to the study by Twijnstra et al. probably there is lack of training in doctors as well as in staff assisting in this type of procedure, thereby limiting the use of laparoscopy for levels 1 and 2 indications [4]. Laparoscopy has not completely become an integral part of training of postgraduates in medical colleges. It should be incorporated with proper training so that better patient management can be done at all levels.

The limitation of our study is that it is a retrospective study done in a single unit and the strength of our study is the adequate number of patients and the changing trends have been clearly evident.

We conclude thereby that definitely the use of laparoscopy has increased significantly in operative procedures and also the indications for laparoscopy have undergone changing trends over the years. Laparoscopic surgery is an economically feasible option nowadays. But, however, the inadequate training at basic levels has still not enabled us to perform skillful laparoscopic surgeries in all centres.

## Figures and Tables

**Table 1:** Number of diagnostic versus operative laparoscopic procedures from 2005 to 2012.

Year	Diagnostic	Percent %	Operative	Percent %	Total
2005	17	62.96	10	37.03	27
2006	25	64.1	14	35.89	39
2007	42	70	18	30	60
2008	36	59.01	25	40.98	61
2009	26	53.06	23	46.94	49
2010	19	42.22	26	57.77	45
2011	19	35.18	35	64.81	54
2012	18	26.08	51	73.91	69

Total	208		196		404

**Table 2:** Indications of laparoscopy during 2005–2012.

Year	Infertility	CPP	AUB	Ovarian Cyst	Uterine anomalies	UV Prolapse	Ectopic Pregnancy	Endometrioma	Others
2005	22	1	1	1	2	—	—	—	—
2006	26	4	2	1	1	1	1	1	2
2007	32	5	6	7	2	—	2	1	5
2008	40	2	7	7	—	1	1	—	3
2009	26	9	2	5	2	1	1	2	1
2010	38	1	1	2	—	1	—	2	—
2011	29	10	7	2	2	3	—	—	1
2012	35	14	12	1	1	3	1	1	1
